# Diagnostic accuracy of GeneReady MTB assay on sputum for detecting pulmonary tuberculosis: a prospective multicenter study in China

**DOI:** 10.1128/jcm.01713-25

**Published:** 2026-06-22

**Authors:** Yuanyuan Shang, Zihan Tang, Yunfeng Deng, Ping Xu, Zichun Ma, Jin Shi, Wei Jing, Jing Xue, Shanshan Li, Liang Li, Yu Pang

**Affiliations:** 1 Department of Bacteriology and Immunology, Beijing Chest Hospital, Capital Medical University/Beijing Tuberculosis and Thoracic Tumor Research Institute 117550 , Beijing, China; 2 Katharine Hsu International Research Centre of Human Infectious Diseases, Shandong Public Health Clinical Centre Affiliated to Shandong University 442536, Jinan, China; 3 Department of Clinical Laboratory, The Fifth People’s Hospital of Suzhou, The Affiliated Infectious Diseases Hospital, Suzhou Medical College of Soochow University 74565, Suzhou, China; 4 Department of Tuberculosis, Beijing Chest Hospital, Capital Medical University/Beijing Tuberculosis and Thoracic Tumor Research Institute Beijing Chest Hospital 117550 , Beijing, China; University of Western Australia, Perth, Australia

**Keywords:** pulmonary tuberculosis, diagnostic, MTB

## Abstract

In this prospective multicenter study, we aimed to evaluate the diagnostic accuracy of the novel GeneReady MTB assay on sputum for the detection of pulmonary tuberculosis (TB). Participants with symptoms suggestive of active pulmonary TB from three Chinese hospitals underwent testing with GeneReady, smear microscopy, mycobacterial culture, and Xpert MTB/RIF (G4) assay. GeneReady demonstrated an analytic limit of detection (LOD) of 23.34 CFU/mL (95% confidence interval [95% CI]: 16.96–43.05). A total of 537 participants meeting the inclusion criteria were included in our analysis. Against a microbiological reference standard, GeneReady had a sensitivity of 92.46% (319/345, 95% CI: 89.02%–94.92%) and a specificity of 96.35% (185/192, 95% CI: 92.33%–98.39%). When stratified by bacterial load, the sensitivity of GeneReady was higher in smear-positive (96.86%) versus smear-negative (87.01%) patients. When compared with Xpert MTB/RIF (G4), GeneReady showed a positive percent agreement of 94.55% (95% CI: 91.48%–96.73%) and a negative percent agreement of 93.24% (95% CI: 88.68%–96.11%). To conclude, GeneReady showed diagnostic accuracy comparable to Xpert MTB/RIF (G4). Its rapid turnaround, lower cost, and ease of use support its potential as an initial diagnostic tool for pulmonary TB in resource-limited settings. Further studies are warranted to evaluate its diagnostic accuracy in clinical settings across different patient populations.

**IMPORTANCE:**

Tuberculosis (TB) remains a global health crisis, hindered by diagnostic gaps in resource-limited settings where high costs restrict access to accurate molecular testing. This study demonstrates that the novel GeneReady MTB assay provides diagnostic accuracy comparable to the widely used Xpert MTB/RIF (G4) assay, but at a significantly lower cost and with simplified operation. By offering a rapid, affordable, and accurate alternative, this test has the potential to expand access to quality TB diagnosis in primary and community healthcare settings, thereby improving case detection and supporting global TB control efforts.

## INTRODUCTION

Tuberculosis (TB), caused by Mycobacterium tuberculosis complex (MTBC), remains a major global public health threat, with an estimated 10.8 million new cases worldwide in 2024 (1–3). A critical gap persists in case detection, as approximately one quarter of incident TB cases are estimated to remain undiagnosed, fueling ongoing transmission. Timely and accurate diagnosis is therefore fundamental to reducing TB-related morbidity and mortality. While MTBC culture has long served as the diagnostic reference standard, its utility is limited by slow turnaround times (several weeks) and substantial laboratory infrastructure requirements (4, 5). Compared with conventional TB diagnostic methods, the molecular-based assays show promise as a fast, sensitive, and specific means for identification of mycobacteria (6–8). Multiple commercial molecular-based techniques have been endorsed by the World Health Organization (WHO) for diagnosis of TB, such as Xpert MTB/RIF (G4 version, Cepheid; Sunnyvale, CA, USA), TB-LAMP (Eiken; Tokyo, Japan), Truenat MTB/RIF (Molbio Diagnostics, Verna, India) (9–14). However, the global scale-up of such technologies, particularly in resource-limited settings, is hampered primarily by high costs associated with instruments and consumables (15–17). There is a pressing need for accurate, rapid, and, crucially, more affordable molecular diagnostic tools that can be deployed closer to the point of care to expand testing access (18, 19).

The GeneReady MTB assay (Life Real, Hangzhou, China) is a novel, integrated cartridge-based molecular test designed to address these barriers of cost and operational complexity, with a streamlined workflow suitable for decentralized laboratory settings. The assay automates nucleic acid extraction and real-time PCR detection within a single-use cartridge, providing results in approximately 80 minutes from decontaminated sputum samples. It is engineered for use in non-specialized laboratory environments, with a per-test cost significantly lower than that of current WHO-recommended molecular assays. To be suitable for decentralized settings, a novel diagnostic must not only be affordable but must also demonstrate diagnostic accuracy comparable to established standards. It is noteworthy that while the newer Xpert MTB/RIF Ultra assay offers enhanced sensitivity, especially in paucibacillary specimens, its adoption in routine clinical laboratories across China during the study period remained limited. Therefore, this study compared GeneReady against the Xpert MTB/RIF (G4) platform, which represents the most widely available molecular diagnostic tool in the participating centers and reflects real-world practice in many resource-constrained settings.

Therefore, we conducted this prospective multicenter study to rigorously evaluate the diagnostic performance of the GeneReady MTB assay against a composite microbiological reference standard and the widely used Xpert MTB/RIF (G4) assay. We hypothesized that GeneReady would demonstrate non-inferior diagnostic accuracy to Xpert MTB/RIF (G4), supporting its potential as a cost-effective alternative for the initial diagnosis of pulmonary TB in resource-constrained settings.

## MATERIALS AND METHODS

### Study design and specimen collection

This prospective multicenter study consecutively recruited individuals with presumptive TB from The Fifth People’s Hospital of Suzhou (marked as site-1), Beijing Chest Hospital (marked as site-2), and Shandong Public Health Clinical Center Affiliated to Shandong University (marked as site-3) between 29 March 2023 and 26 February 2024. Eligible patients met the following criteria: (i) age ≥18 years; (ii) cough duration ≥2 weeks; (iii) ability to provide qualified sputum samples; and (iv) absence of nontuberculous mycobacterial infection. Qualified sputum samples were defined as purulent or mucopurulent specimens of at least 2 mL volume, as assessed visually by trained laboratory staff at each site upon receipt. After enrollment, each participant submitted sputum samples for laboratory analysis. Only one sputum sample per participant was included in the final analysis.

All participants received standardized written information detailing the study objectives and procedures, followed by a formal informed consent process. Written informed consent was obtained prior to enrollment. The study protocol was approved by the institutional ethics committees of all participating hospitals (YLQX-2023-014).

### Analytical sensitivity and specificity of GeneReady

The analytical sensitivity and limit of detection (LOD) for MTB were evaluated according to the following protocol. Reference MTB H37Rv strain (ATCC 27294) was spiked into MTB-free sputum at serial concentrations (1.56–50 CFU/mL) for assay validation. To mimic real-world conditions, the spiked samples were processed using the same NALC-NaOH decontamination procedure as used for clinical specimens. Each dilution level was tested in 20 replicates. The reported LOD₉₅ value of 23.34 CFU/mL refers to the concentration of MTB in the original raw sputum prior to NALC-NaOH treatment. The LOD was defined as the lowest bacterial concentration achieving ≥95% positive, calculated using probit regression analysis. The LOD₉₅ and its 95% confidence interval were derived from the probit model.

### Sputum microbiology

All sputum processing, including decontamination and liquefaction, was performed in accordance with national tuberculosis laboratory biosafety guidelines (initial sputum processing and decontamination were performed under BSL-3 conditions; then, after decontamination, the samples were allowed subsequent molecular testing steps to be conducted under BSL-2 conditions with appropriate personal protective equipment).

Sputum samples were processed in the Clinical Laboratory at each site. The raw sputum was directly smeared onto a slide prior to the addition of NALC-NaOH. Following smear preparation, the remaining approximately 2–5 mL sputum specimen was placed in a centrifuge tube, followed by the addition of two volumes of NALC-NaOH solution. The mixture was then vortexed for 10–20 seconds using a vortex mixer until complete liquefaction of the sputum was achieved. After NALC-NaOH liquefaction, the processed sputum specimen was directly aliquoted for mycobacterial growth indicator tube (MGIT) culture, Xpert MTB/RIF (G4), and GeneReady assays, ensuring all tests were performed on the same specimen. For the MGIT culture, after standing at room temperature for 15 minutes, phosphate-buffered solution is added to the centrifuge tube to bring the total volume to 45 mL. This is followed by centrifugation at 8°C–10°C and 3,000 × *g* for 15–20 minutes. The supernatant is carefully decanted, and the pellet is resuspended in 1 mL of phosphate-buffered solution. Finally, 0.5 mL of the processed sample is inoculated into the MGIT tube.

For smear microscopy, a loopful of raw sputum was stained using the Ziehl–Neelsen (ZN) method and graded according to national guidelines. The stratification in our analysis (“smear-positive” vs “smear-negative”), samples graded as “Positive,” “1+,” “2+,” “3+,” and “4+” were all classified as smear-positive. This is consistent with the common practice in diagnostic studies that use the WHO scale, where “Scanty” and higher grades are typically grouped into the “smear-positive” category for sensitivity analyses. To clarify this, Table 1 summarizes the specific grading rubric used in our study and provides its direct crosswalk to the WHO/IUATLD standard definitions.

**TABLE 1:** Chinese National Guideline Grade crosswalk to the WHO/IUATLD standard definitions

Chinese National Guideline Grade (ZN staining)	Definition	Equivalent WHO/IUATLD Grade
Negative	No acid-fast bacilli (AFB) were observed in 300 distinct consecutive high-power microscopic fields (HPF)	Negative
Positive (with the number of viewed AFB)	1–8 AFB per 300 HPF	Scanty
1+	3–9 AFB per 100 HPF, with continuous observation of 300 HPF	1+
2+	1–9 AFB per 10 HPF, with continuous observation of 100 HPF	2+
3+	1–9 AFB per HPF	3+
4+	≥10 AFB per HPF	3+

The Xpert MTB/RIF assay (G4 version; Cepheid, Sunnyvale, CA, USA) was performed according to the manufacturer’s instructions. This version was selected as it was the standard platform available across all participating sites during the study period. For the Xpert MTB/RIF (G4) assay, a portion of the NALC-NaOH-processed sputum sediment was used. The sediment was washed once with phosphate-buffered saline and resuspended in PBS to the original volume prior to centrifugation. One milliliter of this resuspension was mixed with 2 mL of Xpert MTB/RIF Sample Reagent (provided in the kit), vortexed, and incubated at room temperature for 15 minutes. Subsequently, 2 mL of the mixture was transferred to an Xpert MTB/RIF (G4) cartridge and run on the GeneXpert instrument. Results were reported semi-quantitatively (high, medium, low, very low) based on the cycle threshold of the first positive rpoB probe.

### GeneReady MTB diagnostic assay

**Fig 1:**
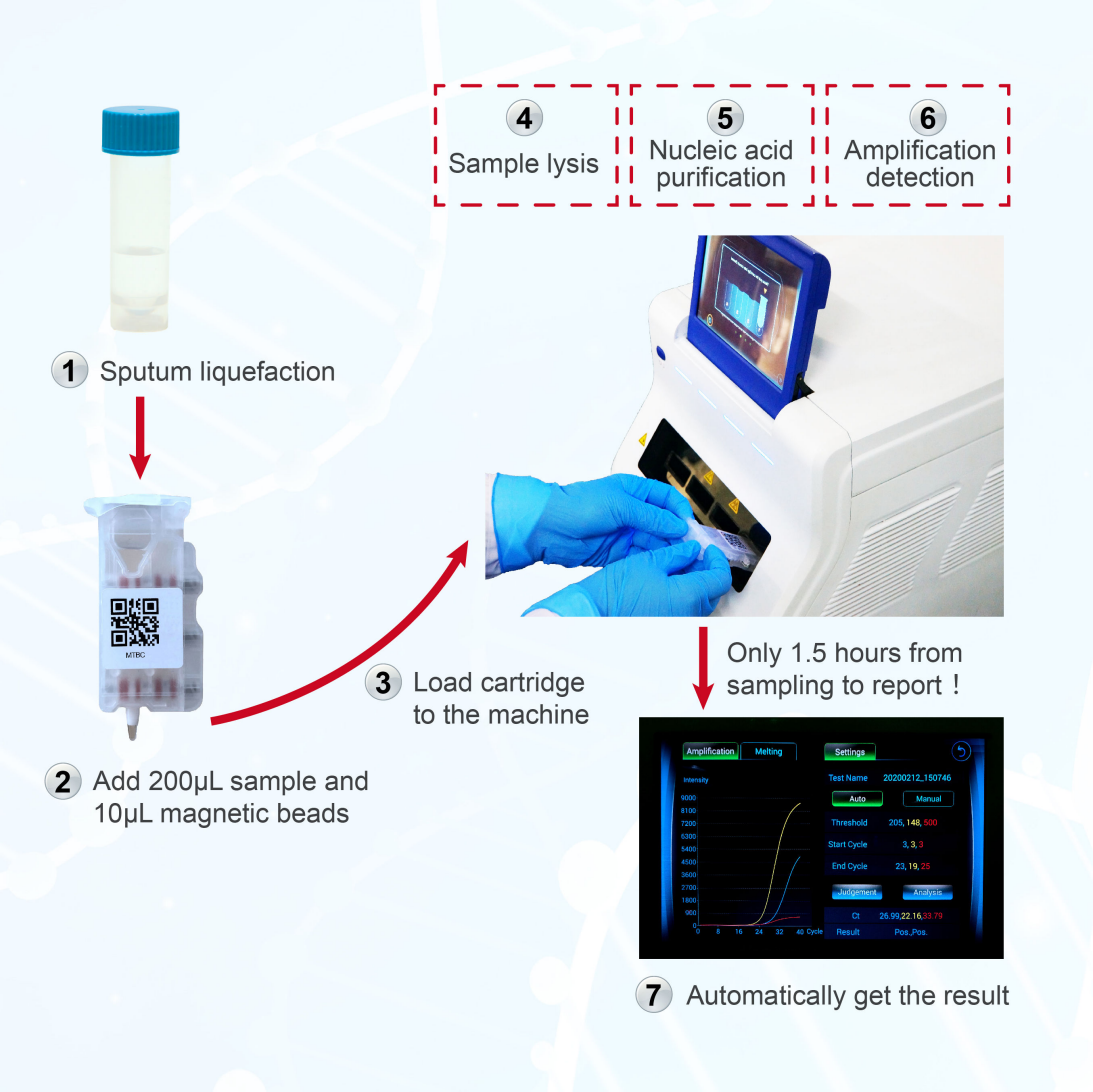
Assay procedure for the GeneReady test.

The GeneReady MTB diagnostic assay employs a fully automated, integrated system for nucleic acid extraction and real-time PCR detection within a single-use microfluidic cartridge. The simplified operation process is shown in Fig. 1. The concentrated sediment was resuspended in phosphate-buffered saline with the original volumes, and 1 mL of the resuspended sediment was mixed with 2 mL of GeneReady sample diluent (C-0005A, Life Real, Hangzhou, China) in a homogenization tube and mixed thoroughly. To initiate the assay, the cartridge lid is opened and the sealing film punctured; 200 μL of the homogenized sample is then added and mixed by pipetting. Subsequently, 10 μL of magnetic beads, which should be ensured to be fully suspended by vortexing before use, are introduced, followed by thorough mixing via repeated aspiration and dispensing (>15 cycles) from multiple positions along the tube wall. The cartridge lid is then closed, and the cartridge is loaded into the Fully Automated Nucleic Acid Testing Analyzer (LifeReady 1000, Life Real, Hangzhou, China). Nucleic acid extraction is achieved through magnetic bead-based purification, whereby bacterial DNA released by lysis is adsorbed onto silica-coated magnetic particles. An automated magnetic rod mechanism transports the beads through a series of wash buffers within the cartridge to remove inhibitors and impurities, after which purified nucleic acids are eluted into the pre-loaded PCR reaction mix. Amplification and detection utilize a multiplex real-time PCR approach targeting two conserved genomic sequences, IS6110 and IS1081, specific to the Mycobacterium tuberculosis complex, with a non-competitive internal control monitored in parallel. Thermal cycling conditions consist of an initial denaturation at 95°C for 5 minutes, followed by 45 cycles of denaturation at 95°C for 15 seconds and combined annealing/extension at 58°C for 30 seconds, with fluorescence acquisition at this step. Detection is based on TaqMan chemistry, with specific probes for IS6110 and IS1081 detected in the FAM and ROX channels, respectively, and the internal control detected in the Cy5 channel. Upon run completion, the integrated software automatically analyzes fluorescence curves and calculates Ct values. A valid result requires the internal control Ct to be ≤38, and samples are interpreted as positive if either or both target channels yield Ct ≤38, ensuring high analytical sensitivity and robust internal quality control throughout the automated process.

To substantiate the claim regarding the assay’s suitability for primary-care settings, the operational profile of the supporting LifeReady 1000 instrument is detailed herein. The analyzer features a compact benchtop footprint (380 × 305 × 343 mm) and a mass of 16 kg, minimizing spatial requirements. Its operational requirements are modest, necessitating a standard power supply (AC 100–240 V, 50/60 Hz) and an ambient temperature of 5°C–40°C with relative humidity ≤85% (non-condensing). The system requires no routine user calibration, as it performs automated self-checks upon initialization, and fluorescence calibration is maintained internally. Maintenance protocols are streamlined, primarily involving external cleaning with 75% ethanol and internal channel decontamination in case of liquid spillage, with no daily maintenance needed. Operator training focuses on pre-analytical steps, such as sputum homogenization, precise pipetting during cartridge loading, and basic instrument operation via the intuitive touchscreen interface. This combination of a small physical footprint, straightforward utility needs, minimal maintenance, and simplified workflow underscores the instrument’s practicality for decentralized laboratory environments with limited resources.

### Quality control and assurance

All participating laboratories underwent standardized training prior to study initiation, rigorous, standardized training covering every step of the GeneReady workflow prior to study initiation. External quality assessment programs for smear, culture, and molecular assays were adhered to throughout the study period. For the GeneReady assay, the kits used in this study were the final commercial version, and lot-to-lot consistency was validated using H37Rv-spiked samples. Both external controls (positive control containing MTB DNA and internal control gene; negative control containing only internal control gene) and an internal control (human RNase P gene, detected in the Cy5 channel) were included in each run to ensure validity and monitor for inhibition.

All MGIT cultures were monitored for contamination, and positive cultures were confirmed as MTBC using the Capilia TB MPT64 Rapid test (Genesis, Hangzhou, China). The contamination rate was 1.02% (6/587). For culture-positive specimens, the mean time-to-positivity was 19.49 days (95% CI: 18.55–20.44). Regular quality control using the H37Rv strain (ATCC 27294) demonstrated consistent performance.

### Statistical analysis

Published data indicated an expected positivity rate of 30% for culture and/or Xpert MTB/RIF (G4) results. At this prevalence, a minimum sample size of 539 participants was required to determine whether the GeneReady assay could achieve 90% sensitivity and 90% specificity with a 5% margin of error. For diagnostic accuracy assessment, a composite microbiological reference standard (MRS) was used as the primary benchmark, defined as positivity in at least one of smear microscopy, MGIT culture, or Xpert MTB/RIF (G4). This approach minimizes the risk of false-negative classification due to culture limitations. When comparing GeneReady with Xpert MTB/RIF (G4), we calculated positive percent agreement (PPA) and negative percent agreement (NPA) rather than sensitivity and specificity, as both tests are molecular assays and neither constitutes a perfect gold standard. The sensitivity of GeneReady for MTB detection was calculated against an MRS. Diagnostic accuracy analyses for GeneReady and comparator tests were performed per patient, with results expressed as point estimates and 95% confidence intervals.

## RESULTS

### Study participants

**Fig 2:**
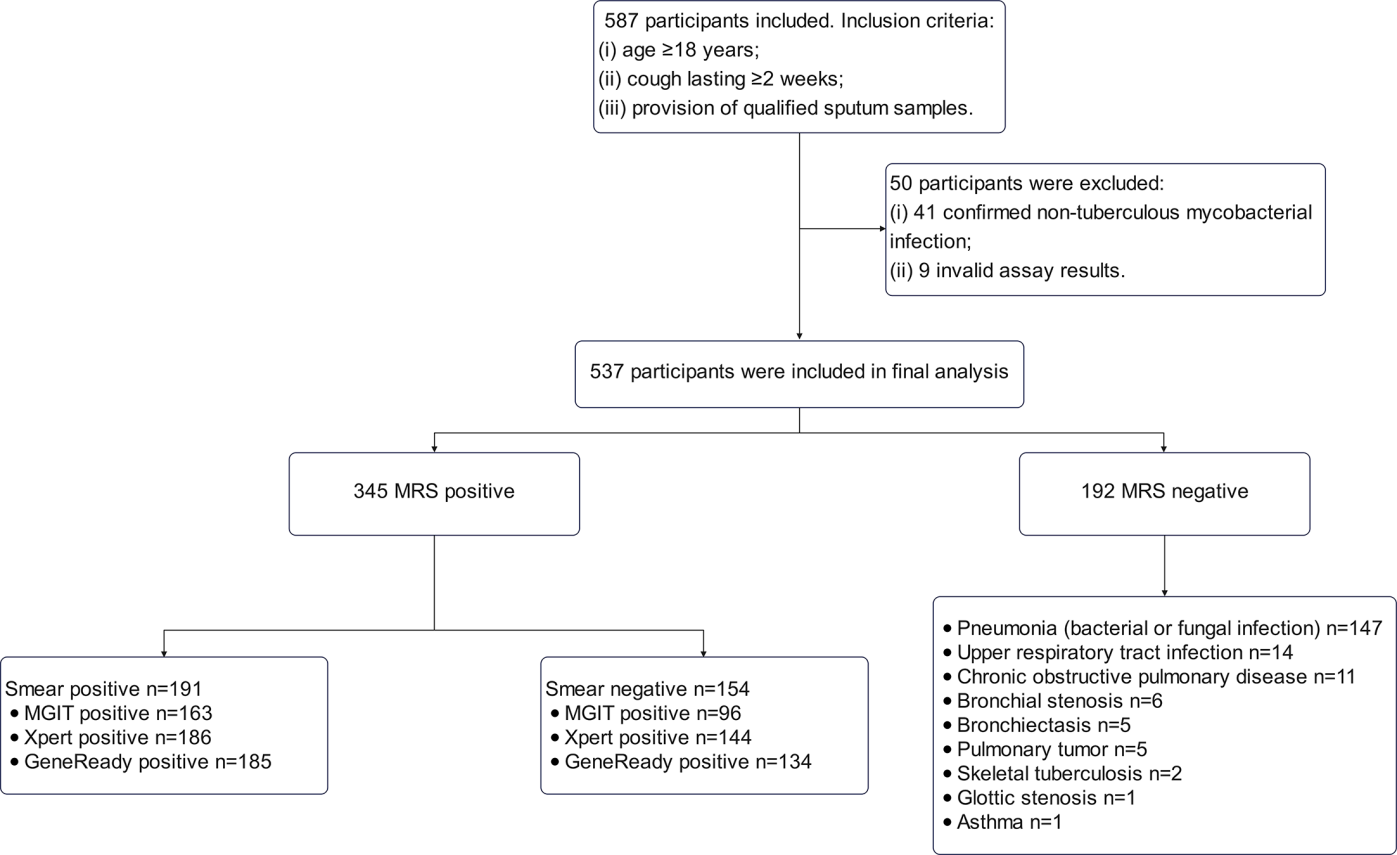
Participants enrollment. MGIT, mycobacterial growth indicator tube. In this study, invalid results include three GeneReady test invalids and six MGIT contamination. GeneReady test results were deemed “invalid” under the following circumstances: internal control (Cy5 channel) Ct value > 38; instrument error (e.g., fluid path anomaly, temperature control failure); significant abnormalities in sample preparation (e.g., sputum not fully liquefied, insufficient sample volume). All invalid samples were retested once. If still invalid, they were deemed a technical failure and excluded from analysis.

In this multicenter study, we recruited 587 participants from three specialized TB public health clinical centers (Fig. 2). Forty-one participants were excluded due to confirmed nontuberculous mycobacteria infection. Nine were excluded for invalid sputum assay results. Among them, six cases had sputum culture contaminated results, and three cases had invalid GeneReady results. The remaining 537 participants were included in the cohort; their sputum samples were collected for laboratory examinations, and assay results were subsequently analyzed. The sample size (*n* = 537) was two participants less than the pre-calculated minimum sample size of 539. According to calculations, this minor shortfall is unlikely to have substantially impacted the statistical power or precision of our findings. Among these 537 participants, 345 were MRS positive, which included 191 smear-positive. And 64.8% (*n* = 348) were male, and 79.5% (*n* = 427) were new cases. Gender distribution across age groups is shown in Table 2. Among the 192 MRS-negative participants, the most common final clinical diagnoses were pneumonia (*n* = 147), upper respiratory tract infection (*n* = 14), chronic obstructive pulmonary disease (*n* = 11), bronchial stenosis (*n* = 6), bronchiectasis (*n* = 5), pulmonary tumor (*n* = 5), skeletal tuberculosis (*n* = 2), glottic stenosis (*n* = 1), and asthma (*n* = 1). This distribution underscores the heterogeneity of non-TB respiratory conditions encountered in our study population (Fig. 2).

**TABLE 2:** Participant demographic characteristics^a^

Demographic characteristics	Male (*n* = 348)	Female (*n* = 189)	Total (*n* = 537)
Average age (years)	52.50 (50.53, 54.47)	44.76 (42.06, 47.45)	49.78 (48.16, 51.39)
Age group (years)			
≤30	57 (16.4)	47 (24.9)	104 (19.4)
31–50	90 (25.9)	65 (34.4)	155 (28.9)
51–70	136 (39.1)	54 (28.6)	190 (35.4)
>70	65 (18.7)	23 (12.2)	88 (16.4)
New cases	276 (79.3)	151 (79.9)	427 (79.5)

^a^ *n *(%), M(P_5_,P_95_).

### LOD and diagnostic accuracy of GeneReady for TB

**Fig 3:**
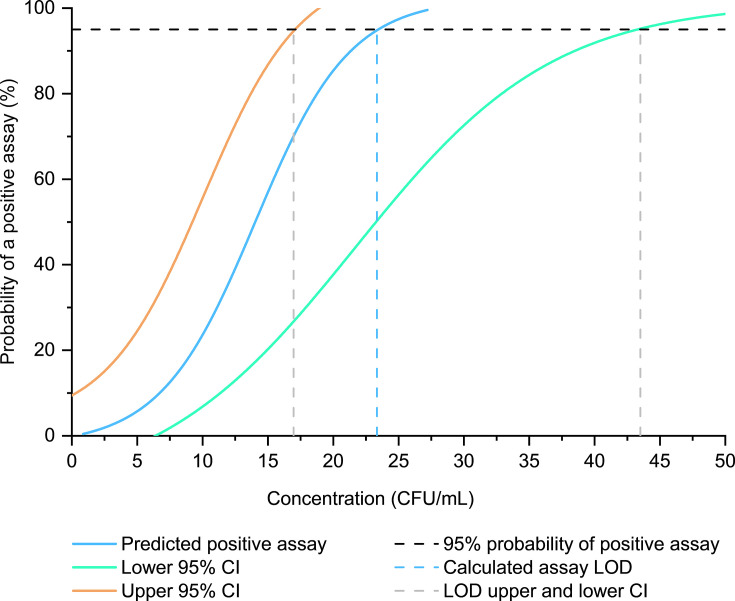
Limit of detection for the reference MTB H37Rv.

The LOD for MTB was determined using statistical probit regression analysis. The LOD₉₅ is defined as the lowest bacterial concentration yielding a 95% detection rate. When the MTB concentration in a sample is ≥23.34 CFU/mL, GeneReady achieves a ≥95% probability of detection (Fig. 3). The associated 95% CI reflects the precision of this estimate, confirming that the true LOD₉₅ value lies between 16.96 and 43.05 CFU/mL with 95% confidence.

Against the MRS, GeneReady demonstrated 92.46% sensitivity (319/345, 95% CI: 89.02%–94.92%; Table 3). Stratification by sputum smear status revealed sensitivities of 96.86% (185/191, 95% CI: 92.97%–98.72%) for smear-positive patients and 87.01% (134/154, 95% CI: 80.42%–91.70%) for smear-negative patients. Specificity relative to MRS reached 96.35% (185/192, 95% CI: 92.33%–98.39%). The assay achieved a positive predictive value (PPV) of 97.85% (319/326, 95% CI: 95.43%–99.06%) and a negative predictive value (NPV) of 87.68% (185/211, 95% CI: 82.29%–91.65%), with an overall concordance rate of 93.85% (504/537, 95% CI: 91.82%–95.89%).

**TABLE 3:** Diagnostic performance of the GeneReady assay for TB using microbiological reference standard and agreement with Xpert MTB/RIF (G4)^*b*^

Reference	Sensitivity/PPA (%, 95% CI)			Specificity/NPA (%, 95% CI)	PPV (%, 95% CI)	NPV (%, 95% CI)	Overall concordance (%, 95% CI)
	All	Smear positive	Smear negative				
Microbiological reference standard^*a*^	319/345 92.46 (89.02, 94.92)	185/191 96.86 (92.97, 98.72)	134/154 87.01 (80.42, 91.70)	185/192 96.35 (92.33, 98.39)	319/326 97.85 (95.43, 99.06)	185/211 87.68 (82.29, 91.65)	504/537 93.85 (91.82, 95.89)
Xpert MTB/RIF (G4)	312/330 94.55 (91.48, 96.73)	182/186 97.85 (94.23, 99.31)	130/144 90.28 (83.93, 94.39)	193/207 93.24 (88.68, 96.11)	312/326 95.71 (92.73, 97.54)	193/211 91.47 (86.64, 94.72)	505/537 94.04 (92.04, 96.04)

^*a*^ Positivity of one of the three tests [sputum smear, sputum culture, sputum Xpert MTB/RIF (G4) for MTB] is defined as a positive microbiological reference standard.

^*b*^ PPA, positive percent agreement; NPA, negative percent agreement; PPV, positive predictive value; NPV, negative predictive value; CI, confidence interval; TB, tuberculosis; MTB, Mycobacterium tuberculosis.

Against Xpert MTB/RIF (G4) (Table 3), GeneReady demonstrated a PPA of 94.55% (312/330, 95% CI: 91.48%–96.73%). Among smear-positive patients, PPA measured 97.85% (182/186, 95% CI: 94.23%–99.31%), while smear-negative patients showed 90.28% PPA (130/144, 95% CI: 83.93%–94.39%). NPA against Xpert MTB/RIF (G4) stood at 93.24% (193/207, 95% CI: 88.68%–96.11%). Further analysis yielded a PPV of 95.71% (312/326, 95% CI: 92.73%–97.54%) and NPV of 91.47% (193/211, 95% CI: 86.64%–94.72%), culminating in 94.04% overall concordance (505/537, 95% CI: 92.04%–96.04%).

**Fig 4:**
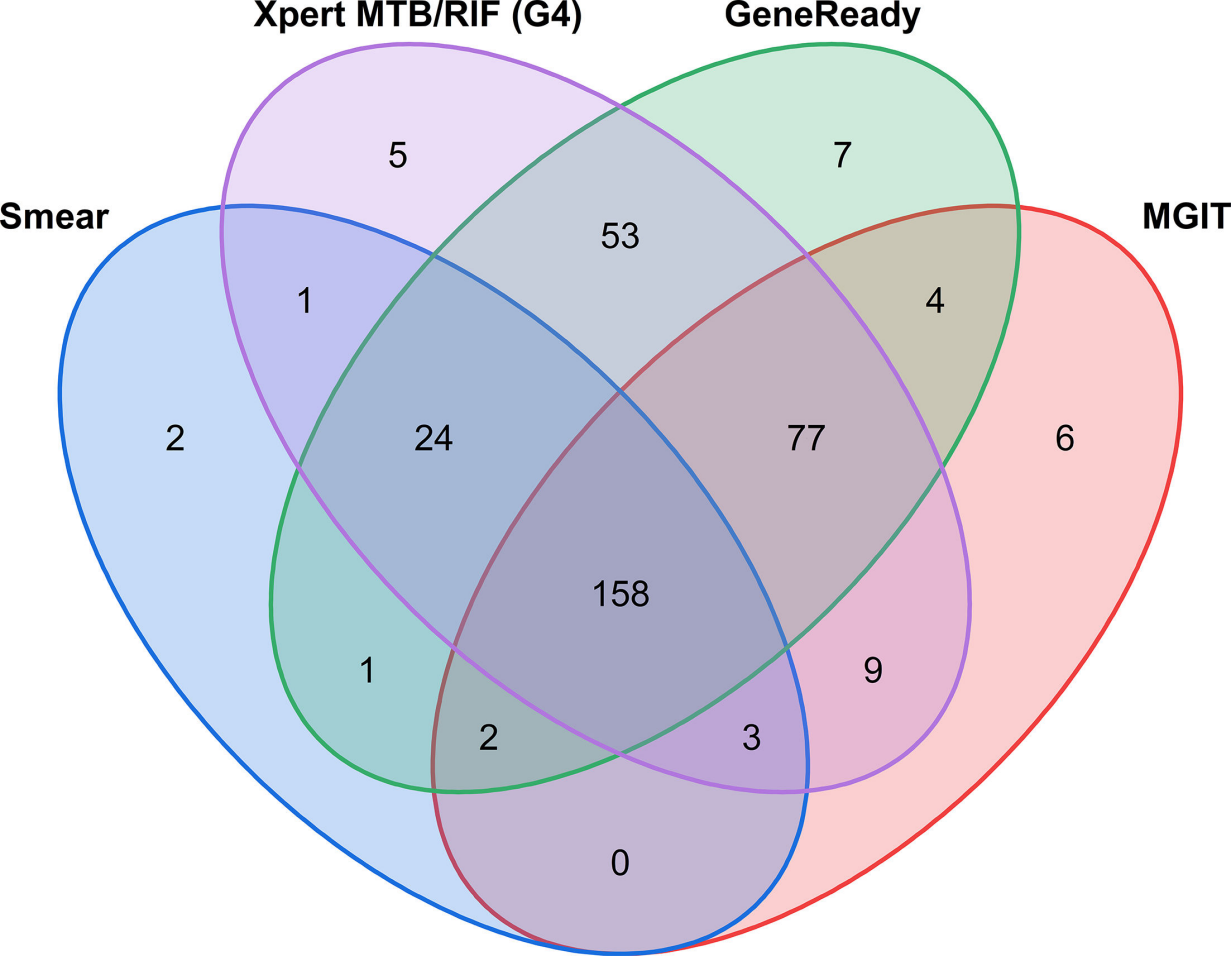
Venn diagram of MTB detection positive overlap by GeneReady, Xpert MTB/RIF (G4), smear, and MGIT culture in sputum samples.

The distribution of positive specimens detected by smear, Xpert MTB/RIF (G4), MGIT, and GeneReady is shown in Fig. 4. Most Xpert MTB/RIF (G4)-positive (312/330) and MGIT-positive (245/259) cases were also positive by GeneReady. Additionally, smear, Xpert MTB/RIF (G4), MGIT, and GeneReady exclusively detected 2, 5, 6, and 7 positive cases, respectively. A total of 158 cases were positive by all four detection methods.

### Site-level diagnostic performance of GeneReady assay

To assess the operational consistency and potential heterogeneity across different study sites, we performed a site-specific analysis of the GeneReady assay’s diagnostic accuracy. As shown in Table 4, the sensitivity against the microbiological reference standard ranged from 86.87% (95% CI: 78.24%–92.55%) at site-2 (Beijing) to 95.65% (95% CI: 89.65%–98.39%) at site-1 (Suzhou), with site-3 (Shandong) achieving 93.89% (95% CI: 87.93%–97.13%). Specificity was consistently high across all sites, ranging from 91.23% (95% CI: 79.96%–96.73%) at site-2 to 98.73% (95% CI: 92.18%–99.93%) at site-3. Overall concordance rates were 96.49% (95% CI: 93.73%–99.25%), 88.46% (95% CI: 83.45%–93.48%), and 95.71% (95% CI: 92.97%–98.45%) at sites 1, 2, and 3, respectively.

**TABLE 4:** The GeneReady MTB assay diagnostic performance in site-level compared to MRS

Site	Sample size	Sensitivity (%, 95% CI)	Specificity (%, 95% CI)	PPV (%, 95% CI)	NPV (%, 95% CI)	Overall concordance (%, 95% CI)
1	171	95.65 (89.65, 98.39)	98.21 (89.19, 99.91)	99.10 (94.36, 99.95)	91.67 (80.88, 96.89)	96.49 (93.73, 99.25)
2	156	86.87 (78.24, 92.55)	91.23 (79.96, 96.73)	94.51 (87.06, 97.96)	80.00 (67.88, 88.52)	88.46 (83.45, 93.48)
3	210	93.89 (87.93, 97.13)	98.73 (92.18, 99.93)	99.19 (94.93, 99.96)	90.70 (82.00, 95.61)	95.71 (92.97, 98.45)

**Fig 5:**
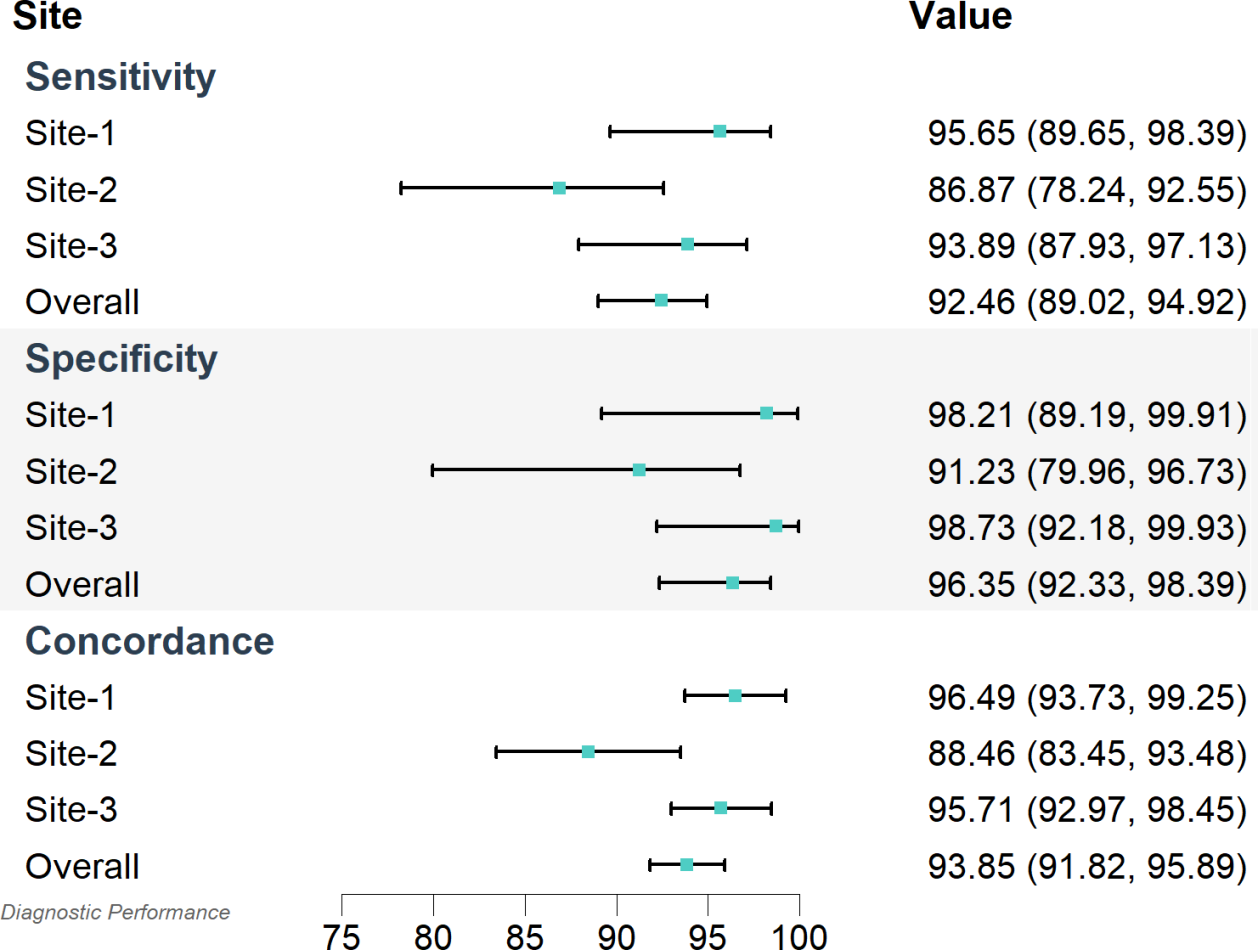
Forest plots of sensitivity, specificity, and concordance rates across different sites.

The heterogeneity in performance across sites was visually assessed using forest plots (Fig. 5). Although point estimates showed some variation, particularly for sensitivity at site-2, the 95% confidence intervals of all three sites overlapped substantially for sensitivity, specificity, and overall concordance metrics. This overlapping of confidence intervals suggests that the observed differences are likely due to random variation rather than systematic operational heterogeneity, especially considering the relatively small sample size at each site for subgroup analysis.

### Performance of GeneReady stratified by Xpert MTB/RIF (G4) semi-quantitative categories

We further stratified the performance of GeneReady according to the semi-quantitative bacterial load categories reported by Xpert MTB/RIF (G4). Among the 330 Xpert MTB/RIF (G4)-positive samples, distribution across categories was as follows: 65 “very low,” 125 “low,” 106 “medium,” and 34 “high.” GeneReady detected 54/65 (83.08%, 95% CI: 71.7–90.8) in the “very low” category, 122/125 (97.60%, 95% CI: 92.9–99.4) in “low,” 102/106 (96.23%, 95% CI: 90.5–98.9) in “medium,” and all 34/34 (100%, 95% CI: 89.7–100.0) in “high” (Table 5). The sensitivity of GeneReady demonstrated a clear correlation with bacillary burden, maintaining high performance down to the “low” category and exhibiting a modest decline only in the “very low” group.

**TABLE 5:** Comparison of Xpert semi-quantitative results with GeneReady results

Xpert MTB/RIF (G4) semi-quantitative category	Xpert MTB/RIF (G4) semi- quantitative category (*n*)	GeneReady positive samples (*n*)	Sensitivity of GeneReady (%, 95% CI)
Very low	65	54	83.08 (71.7, 90.8)
Low	125	122	97.60 (92.9, 99.4)
Medium	106	102	96.23 (90.5, 98.9)
High	34	34	100.00 (89.7, 100.0)

### Individual-level comparison with Xpert MTB/RIF (G4) and MGIT culture

To further characterize the diagnostic performance of GeneReady, pairwise comparisons with Xpert MTB/RIF (G4) and MGIT culture were performed at the individual participant level (Table 6). Among the 537 participants, 312 were positive by both GeneReady and Xpert, and 193 were negative by both assays. Discordant results were observed in 32 cases, including 18 that were Xpert positive but GeneReady negative and 14 that were Xpert negative but GeneReady positive. Of the 18 Xpert positive cases missed by GeneReady, 11 (61.1%) were classified as very low semiquantitative category by Xpert, suggesting that the lower input volume of GeneReady, equivalent to 22 μL of raw sputum compared with 222 μL for Xpert, may limit its detection sensitivity in paucibacillary specimens. The 14 Xpert-negative but GeneReady-positive cases may reflect false-negative Xpert results or true positives missed by Xpert, potentially attributable to its semiquantitative threshold or rare PCR inhibition.

**TABLE 6:** Individual-level comparison of GeneReady results with Xpert MTB/RIF (G4) and MGIT culture

Comparator	Comparator result	GeneReady positive	GeneReady negative	Total
Xpert MTB/RIF (G4)	Positive	312	18	330
	Negative	14	193	207
MGIT culture	Positive	241	18	259
	Negative	85	193	278

In the comparison with the MGIT culture, 241 cases were positive by both methods, and 193 were negative by both. Discordant results were identified in 103 cases, comprising 18 that were culture positive but GeneReady negative and 85 that were culture negative but GeneReady positive. Among the 18 culture-positive cases missed by GeneReady, 15 (83.3%) were smear negative, further indicating that low bacillary load was the primary reason for false negativity. The 85 culture-negative but GeneReady-positive cases most likely represent false negative culture results caused by loss of mycobacterial viability during decontamination or overgrowth of contaminants, both well-recognized limitations of culture-based methods. This interpretation is supported by the finding that 78 of these 85 cases (91.8%) had a positive result by either Xpert or smear microscopy, confirming the presence of tubercle bacilli.

These individual-level comparisons demonstrate that GeneReady results are highly concordant with both Xpert and MGIT culture, and the majority of discordant findings occurred in specimens with very low bacterial loads, a challenge common to all molecular assays. The additional detection of culture-negative cases underscores the potential of GeneReady to complement or even replace culture in certain settings, particularly where rapid turnaround and operational simplicity are prioritized.

## DISCUSSION

The scale-up of molecular diagnosis is essential to facilitate continued progress toward tuberculosis elimination. In the present study, we first developed a novel GeneReady MTB assay to identify MTBC from sputum specimens within 80 minutes. The multicenter diagnostic accuracy study indicated that the rapid molecular GeneReady assay had overall comparable performance characteristics to Xpert MTB/RIF (G4), yielding a sensitivity of 92.46% and a specificity of 96.35%. It is important to clarify that the diagnostic accuracy of GeneReady was assessed against a composite microbiological reference standard, whereas the comparison with Xpert MTB/RIF (G4) was conducted as an agreement analysis (positive and negative percent agreement), reflecting that both are molecular tests and neither constitutes a perfect gold standard. A key methodological difference is the effective volume of raw sputum tested. The Xpert MTB/RIF (G4) assay tests an equivalent of approximately 222 μL of raw sputum. In contrast, the GeneReady assay uses 200 μL of liquefied sputum, which corresponds to only about 22 μL of the original raw sample (due to its 1:2 dilution step). This difference in effective input volume may partly explain the trend toward slightly higher sensitivity for Xpert in some comparisons.

Using MRS as the gold standard, another study showed that the Truenat MTB test was found to have sensitivities of 73.0% and 79.5% in the microscopy center and reference laboratory, respectively (13), which was remarkably lower than GeneReady. Additionally, due to the close nature of the assay, the GeneReady MTB assay presents a lower risk of laboratory contamination than the Truenat assay, especially in resource-limited regions. Taken together, these results highlight that GeneReady could be considered as initial tests for the diagnosis of active TB in primary healthcare facilities. Further validation studies are required to determine its performance in microscopy centers.

An important consideration for implementing novel diagnostic assays in diverse healthcare settings is the consistency of performance across different operational environments. Our site-level analysis revealed that GeneReady maintained high diagnostic accuracy across all three participating centers, with overlapping 95% confidence intervals for sensitivity, specificity, and overall concordance (Table 4 and Fig. 5). The observed variation in point estimates, particularly the relatively lower sensitivity at site-2 (Beijing Chest Hospital, 86.87% vs 95.65% at site-1), warrants contextual interpretation. As a leading national referral center for tuberculosis in China, Beijing Chest Hospital receives a substantial number of complex and diagnostically challenging cases referred from across the country. This tertiary care setting naturally includes a higher proportion of paucibacillary cases, treatment-experienced patients, and individuals with comorbidities that may affect bacillary load and test performance. Indeed, our supplementary analysis confirmed that site 2 had the highest proportion of smear-negative patients among microbiologically confirmed cases (51.6%), compared with site 1 (31.5%) and site 3 (40.6%). This difference in case mix likely contributed to the lower point estimate of sensitivity observed at this center compared to others. The rigorous patient selection at this specialized center, where many cases represent diagnostic dilemmas unresolved at primary or secondary care levels, likely contributed to the observed sensitivity estimate. Importantly, despite this more challenging case mix, the assay still achieved high specificity (91.23%) and overall concordance (88.46%), and the confidence intervals overlapped substantially with those of the other sites, indicating no statistically significant heterogeneity. These findings collectively suggest that GeneReady performs robustly across diverse clinical settings, including specialized referral centers with complex patient populations, supporting its potential for implementation across different levels of the healthcare system.

A relevant consideration for policy audiences is the performance of GeneReady relative to the newer Xpert MTB/RIF Ultra (Ultra) assay, which exhibits higher sensitivity, particularly in smear-negative and paucibacillary tuberculosis. While a direct head-to-head comparison with Ultra was not feasible in this study due to its limited availability in Chinese clinical laboratories at the time of enrollment, our data can be contextualized through published literature. Studies have demonstrated that Ultra improves sensitivity by approximately 5%–10% over Xpert MTB/RIF (G4) (20–22). In our cohort, GeneReady achieved a sensitivity of 92.46% overall and 87.01% in smear-negative patients against the MRS, which is comparable to the reported performance of Xpert MTB/RIF G4 in similar populations. This suggests that GeneReady may not reach the same detection threshold as Ultra in very low bacterial-load specimens, a trade-off that must be weighed against its substantially lower cost and operational simplicity. Nevertheless, GeneReady’s sensitivity in smear-negative cases remains markedly superior to that of smear microscopy, supporting its utility as an initial diagnostic tool in settings where Ultra is not yet accessible. Future studies should prioritize direct comparison with Ultra in diverse patient populations to further clarify the assay’s role in the evolving diagnostic landscape.

In line with previous findings, the sensitivity of GeneReady was lower in smear-negative individuals, reflecting a significant impact of airway bacillary burden on test sensitivity. Notably, the sensitivity of GeneReady shows a clear and expected correlation with bacterial load when examined at a granular level, which is similar to the sensitivity of Xpert MTB/RIF (G4) (Table 7). Moreover, the decline in sensitivity for GeneReady when moving from smear-positive to smear-negative patients (96.86% to 87.01%) was less pronounced. Of note, we used direct smear microscopy with ZN staining. Direct examination without culture enrichment inherently misses many low-bacillary samples, and ZN staining has limited sensitivity in paucibacillary specimens. Furthermore, the relatively low positivity rate of MGIT culture in our cohort (259/537, 48.2%) may have led to an underestimation of the true number of culture-confirmed, smear-negative TB cases. This potential underestimation in the denominator of the microbiological reference standard-positive, smear-negative group would, by definition, contribute to the higher calculated sensitivity of molecular assays like GeneReady in this subgroup. Together, these factors likely contributed to the low smear-positive rate and the high proportion of molecular-positive results among smear-negative patients. This relatively high sensitivity in smear-negative patients is likely attributable to the assay’s high analytical sensitivity (LOD₉₅: 23.34 CFU/mL) and its stable nucleic acid extraction efficiency from low bacterial-load specimens.

**TABLE 7:** Sensitivity of GeneReady and Xpert MTB/RIF (G4) in different sputum smear grading results

Smear grade	Equivalent WHO grade	Number of patients (*N*)	GeneReady sensitivity % (*n*/*N*)	Xpert MTB/RIF sensitivity % (*n*/*N*)
Negative	Negative	346	40.75 (141/346)	41.62 (144/346)
Positive	Scanty	13	92.31 (12/13)	84.62 (11/13)
1+	1+	81	96.30 (78/81)	100.00 (81/81)
2+	2+	50	98.00 (49/50)	94.00 (47/50)
3+	3+	32	96.88 (31/32)	100.00 (32/32)
4+	3+	15	100.00 (15/15)	100.00 (15/15)
All smear-positive	/^*a*^	191	96.86 (185/191)	97.38 (186/191)
Smear-negative and MRS-positive	/	154	87.01 (134/154)	93.51 (144/154)

^*a*^ “/” in this table indicates that the indicator is not applicable.

Our stratified analysis by Xpert MTB/RIF (G4) semi-quantitative categories further elucidates the relationship between bacterial load and GeneReady detection capability (Table 5). The assay achieved near-perfect sensitivity in “low,” “medium,” and “high” categories (97.6%–100%), indicating robust performance across most clinically relevant bacillary loads. In the “very low” category, sensitivity remained substantial at 83.1%, which reflects the inherent challenge of detecting paucibacillary specimens common in smear-negative and early-stage TB. This gradual decline in sensitivity with decreasing bacterial burden mirrors patterns observed with other molecular assays, including Xpert MTB/RIF (G4), and underscores the importance of complementary diagnostic strategies in low-load settings. Nevertheless, the high sensitivity maintained even in low-burden samples supports GeneReady’s suitability for routine use in settings where smear-negative TB is prevalent.

Our results extend this observation to MGIT culture, where sensitivity was lower in smear-negative than in smear-positive individuals. Besides, the sensitivity of MGIT was lower than Xpert MTB/RIF (G4) and GeneReady, especially in individuals with paucibacillary TB. This finding might suggest the overexposure of samples to the decontamination reagents, resulting in the increased risk of false-negative cultures due to loss of mycobacterial viability. The observed low contamination rate (1.02%) in our study further supports this interpretation, as it indicates effective but potentially harsh decontamination that may have reduced mycobacterial viability, particularly in paucibacillary specimens. Our previous experimental study demonstrated that the sputum quality had a greater impact on MGIT culture results than on Xpert MTB/RIF (G4) assay results (23), arising from the distinct inherent operational workflows of the two methodologies. Therefore, the sputum quality may be another potential explanation for the impaired sensitivity of MGIT culture in our analysis. Our finding has important practical implications. Given the inherent risk of false-negative results associated with complicated manual procedures in conventional culture, molecular diagnostics offer superior performance for TB detection, especially at primary healthcare laboratories.

The operational complexity of the GeneReady assay should be considered within the updated WHO target product profile framework for peripheral tuberculosis detection. This framework distinguishes near point-of-care tests from low-complexity assays. Near point-of-care tests are instrument-based and require only basic pipetting skills without the need for a laboratory. Low-complexity assays need peripheral laboratory infrastructure. The GeneReady assay meets several near point-of-care criteria. It uses a compact analyzer and requires minimal infrastructure with approximately five minutes of hands-on time. However, the manual magnetic bead mixing step demands more precision than the basic pipetting envisioned for optimal near point-of-care tests. Therefore, the current version aligns more closely with the low-complexity category. Importantly, other platforms like Truenat have similar operational characteristics and are recommended by the WHO for use in microscopy centers. WHO modeling supports that operational simplicity and expanded access can appropriately balance against full automation when the priority is increasing testing coverage in resource-limited settings. Future versions of the GeneReady assay could incorporate pre-analytical automation to better align with optimal near point-of-care criteria.

We also acknowledged several obvious limitations to the present study. Firstly, the GeneReady assay is designed as a streamlined test; however, its sample preparation still involves manual steps, such as vortexing and repeated pipette mixing with magnetic beads. As such, procedural variations could theoretically influence assay specificity; we implemented rigorous, standardized training covering every step of the workflow across all sites. The high and consistent specificity observed (96.35% overall; site-level range 91.23%–98.73%) suggests that under controlled conditions, this impact was minimal. These procedures require technical consistency from the operator and may compromise standardization and reproducibility in resource-limited primary care laboratories, and these preprocessing requirements currently position GeneReady as suitable for decentralized laboratories rather than true point-of-care testing. Future versions that integrate or automate these preprocessing steps would significantly improve ease of use and suitability for near-patient settings. Similarly, the multi-step pretreatment for MGIT culture, including PBS washing, resulted in an effective inoculation volume of only 200–250 μL of original sputum. What’s more, samples underwent standard NALC-NaOH decontamination before MGIT culture. While necessary to prevent overgrowth by commensals, this step can reduce mycobacterial viability, especially in paucibacillary specimens. This marked reduction in input sample volume and the preprocessor may explain the relatively lower MGIT culture positivity rate observed in this study (4). Second, the final sample size (*n* = 537) was two participants less than the pre-calculated minimum sample size of 539. Although this minor shortfall is unlikely to have substantially impacted the statistical power or precision of our findings, it should be noted as a limitation. Third, all three participating sites were tertiary referral hospitals, which may not fully represent the diversity of laboratory settings at peripheral or district levels. While our study demonstrated consistent performance across three specialized centers, each center’s sample size for subgroup analysis was relatively limited (156–210 participants). Although we observed overlapping confidence intervals, suggesting no significant heterogeneity, larger multi-center studies with broader representation of primary healthcare settings would provide more definitive evidence regarding the assay’s operational robustness in truly resource-limited environments. Fourth, while Xpert MTB/RIF Ultra represents an improved reference standard with higher sensitivity, it was not available for inclusion in this study. Our comparison with Xpert MTB/RIF (G4) reflects the diagnostic reality in many resource-limited laboratories but may not fully represent performance against the latest ultra-sensitive molecular assays. We have attempted to bridge this gap through literature-based sensitivity analysis, yet direct comparative data are warranted in future research. Fifth, the LOD determination in this study employed the reference strain H37Rv and probit regression analysis following CLSI EP17-A2; however, we did not use the National Institute for Biological Standards and Control (NIBSC) reference standard recommended by WHO. Although H37Rv is a widely accepted surrogate, direct comparison with NIBSC-based LOD values may be limited. Future studies should consider incorporating NIBSC standards to enhance international comparability. Sixth, the known heterogeneity in performance is frequently seen across populations with different disease spectrums, such as children and HIV-infected populations (24–26). Therefore, more work is needed to evaluate the diagnostic accuracy in clinical settings across different patient populations. Finally, it is important to acknowledge that the GeneReady assay is designed for the detection of MTBC alone and does not identify drug resistance. This characteristic implies that a positive result necessitates a reflex testing pathway, wherein samples must be forwarded for subsequent drug susceptibility testing (DST). This additional step introduces operational complexity, increases costs, and may delay the initiation of appropriate therapy for patients with drug-resistant tuberculosis (DR-TB), presenting a potential clinical risk, particularly in settings with a high prevalence of DR-TB. However, this limitation is of lesser programmatic concern when the assay is applied within its intended use case: large-scale active case-finding and screening among presumptive TB populations where the pre-test probability of drug resistance is low. A prime example is the rapid screening of close contacts of known TB patients. In this context, the overriding public health priority is the high-throughput and cost-effective identification of any MTB infection to interrupt transmission chains swiftly. A positive result promptly triggers necessary epidemiological investigations and enrolls the individual into the clinical care pathway, at which point DST can be arranged as a sequential procedure. Thus, the operational simplicity and favorable cost profile of the GeneReady assay render it highly suitable for such mass screening initiatives. In these scenarios, the inability to concurrently detect drug resistance represents a balanced and pragmatic trade-off for achieving the primary objective of rapid, widespread TB case detection.

The performance of the GeneReady MTB assay was further contextualized by benchmarking its key operational characteristics against the WHO target product profile (TPP) criteria for high-priority target product profiles for new tuberculosis diagnostics tests (Table 8). The assay’s sensitivity (92.46%) and specificity (96.35%) against the microbiological reference standard exceed the minimal TPP thresholds (≥80% and ≥98%, respectively), while its specificity approaches the optimal target (≥98%). Its turnaround time of approximately 80 minutes falls between the minimal (<2 hours) and optimal (<20 minutes) TPP criteria, positioning it as a rapid but not ultra-rapid test. Biosafety requirements are the same as Xpert MTB/RIF (G4), which may present a logistical challenge in some low-resource settings. Cost constitutes a pivotal barrier to the global scale-up of molecular diagnostics for TB. With an estimated per-test cost of about US $5.00, which is a preliminary vendor-provided estimate, the assay aligns with the minimal TPP price target (<$6.00) and is considerably lower than current market prices for Xpert MTB/RIF (G4) cartridges ($9.98). The accompanying instrument (LifeReady 1000 Analyzer) is priced at approximately RMB 65,000 (about US$9,000), which is considerably lower than the capital cost of a GeneXpert module. This lower instrument price, combined with the low per-test cartridge cost, makes the GeneReady system an economically viable option for primary and community healthcare facilities with limited budgets. Overall, this alignment demonstrates that GeneReady meets or approaches the minimal TPP standards for sensitivity, specificity, time-to-result, and cost, supporting its potential as an affordable, accurate, and rapidly deployable tool for initial TB diagnosis in resource-constrained settings. Its performance profile is particularly well-suited for high-throughput screening and active case-finding programs where cost-effectiveness and operational simplicity are prioritized over ultra-rapid turnaround or concurrent drug-resistance detection.

**TABLE 8:** Performance of the GeneReady MTB assay compared to WHO TPP criteria for initial tuberculosis diagnostic tests^a^

Characteristic	Optimal requirement	Minimal requirement	GeneReady performance
Sensitivity	>95% (compared to culture)	≥80% (compared to culture)	92.46 (89.02, 94.92)
Specificity	≥98% (compared to culture)	≥98% (compared to culture)	96.35 (92.33, 98.39)
Time to result	<20 minutes	<2 hours	80 minutes
Biosafe	Should have the same requirements as Xpert MTB/RIF	Same as smear microscopy	Same requirements as Xpert MTB/RIF
Price of individual test	<$4.00	<$6.00	$5.00

^a^ The cost figures represent the estimated price of the test cartridge only and do not include expenses for sample pre-processing, equipment amortization, maintenance, or personnel time. The Xpert MTB/RIF (G4) cartridge price (US $9.98) reflects negotiated public-sector pricing. A direct comparison of cartridge prices does not account for the additional value of rifampicin resistance detection provided by Xpert MTB/RIF (G4), which is not offered by GeneReady.

To conclude, this multicenter diagnostic accuracy study indicates that the rapid molecular GeneReady assay shows overall comparable performance to the widely implemented Xpert MTB/RIF (G4) assay. The operational simplicity and low-cost disposable cartridge collectively support its feasibility for implementation in resource-limited settings. It should be noted, however, that both the GeneReady and Xpert MTB/RIF (G4) cartridge prices quoted in this study represent the cost of the disposable cartridge only, and do not include pre-processing reagents, equipment, or personnel costs. Moreover, Xpert MTB/RIF (G4) provides additional clinical value by detecting rifampicin resistance, a feature not captured in a simple price comparison. And more work is needed to evaluate its diagnostic accuracy in clinical settings across different patient populations. While future studies should evaluate its performance against Xpert MTB/RIF Ultra in paucibacillary populations, the current findings support its potential as an accessible, cost-effective tool for initial TB diagnosis in resource-constrained settings where Ultra is not yet routinely available.

## Data Availability

The statistical data supporting the findings are presented within the article. Individual-level patient data are not publicly available due to privacy and ethical restrictions but may be obtained from the corresponding author upon reasonable request, subject to institutional review board approval. The GeneReady reagent kit is available from the corresponding author for research purposes upon request. A material transfer agreement (MTA) may be required.

## SUPPLEMENTAL MATERIAL

File S1 jcm.01713-25-s0001.docx The technical appendix for GeneReady includes details on key reagents, methodology, testing procedures, and interpretation methods.

ASM does not own the copyrights to Supplemental Material that may be linked to, or accessed through, an article. The authors have granted ASM a non-exclusive, world-wide license to publish the Supplemental Material files. Please contact the corresponding author directly for reuse.
