# Supplementary material for: Diagnostic accuracy of GeneReady MTB assay on sputum for detecting pulmonary tuberculosis: a prospective multicenter study in China
Source: J Clin Microbiol. 2026 Jun 22;64(8):e01713-25. doi: 10.1128/jcm.01713-25 (PMC13463822; doi:10.1128/jcm.01713-25)
Supplement: Supplementary file 1 [file jcm.01713-25-s0001.docx]

**Technical appendix of GeneReady MTB diagnostic assay**

**Main ingredients**

Supplementary Table 1. The main ingredients of GeneReady MTB detection kit.

| Component name | Specifications | Quantity | Main components |
| --- | --- | --- | --- |
| MTBC test cartridge | 1 Per person | 24 individual | Lysis buffer (guanidinium isothiocyanate, sodium dodecyl sarcosinate, Triton X-100, polyethylene glycol, sodium acetate), Wash 1 (Tris-HCl, potassium chloride, polyethylene glycol), Wash 2 (Tris-HCl, potassium chloride, polyethylene glycol, blocking agent), primer probe solution, PCR reaction mixture (tris-hydroxymethyl glycine, potassium acetate, magnesium chloride, dNTPs, Taq DNA polymerase), and auxiliary components such as silicone oil and paraffin. |
| Magnetic bead | 280 μL per tube | 1 tube | Magnetic microsphere suspension |
| MTBC negative control | 1600 μL per tube | 1 tube | RNase P Inactivation Engineering Strain Containing Cloning Recombinant Internal Standard Gene |
| MTBC positive control | 1600 μL per tube | 1 tube | RNase P and ZJTBP4 Inactivated Engineering Strains Containing Cloning Recombinant Internal Standard Gene and Mycobacterium Tuberculosis Genome DNA |

*Note: Components of reagent kits with different batch numbers are not interchangeable. It is recommended to use the sample diluent (C0005-A, Zhejiang Hangzhou Medical Device Approval No.20210442) produced by Life Real, Hangzhou, China. for liquefaction of sputum samples.

**Storage conditions and expiration date**

2-8℃, light proof storage, shelf life 12 months. The date of manufacture and expiry date are indicated on the label. The temperature inside the box must be maintained at 2~8℃ during transportation. Ice packs may be used. Avoid placing the kit below 0℃ .

**Applicable instruments**

This kit is compatible with the fully automated nucleic acid testing analyzers (model: LifeReady 1000) from Life Real, Hangzhou, China and the fully automated nucleic acid testing analysis system (model: LifeReady 1000 XVI) from Life Real, Hangzhou, China.

**Sample Requirements**

1. Sample type: human sputum.

2. Sample collection: Collect sputum coughed out by the patient into a wide-mouth container and send it for examination in a sealed manner, taking precautions to prevent contamination and drying. For specific collection methods, please refer to the "Clinical Technical Operation Specifications (TB Volume)" or the "TB Laboratory Testing Procedures".

3. Transportation: After collection, regardless of the transportation distance, packaging and labeling should be carried out as required. Transportation can be c

nducted using ice-cold containers or foam boxes with ice sealing. Avoid inversion and strictly prevent sputum leakage.

4. Storage: samples that cannot be tested on the same day should be stored at 2-8℃ for no more than 72 hours, at-20±5℃ for no more than 90 days, and at-70℃ or below for no more than 12 months, but should avoid repeated freeze-thaw more than 3 times.

**Method of calibration**

1. Preparation Before Testing

(1) Prepare the instrument: Turn on the power. After the login screen appears, click [Login]. The instrument will complete self-check and be ready for use.

(2) New program: For first-time users, click [New Experiment] on the instrument interface. After the interface changes, select [Scan QR Code] to scan the program QR code on the side of the kit packaging.

Align with the instrument's scanning module and initiate the [MTBC1] scanning program. Once [MTBC1] is successfully created (this step will not interfere if the program is already stored in the instrument), exit the interface. The [MTBC1] detection procedure is as follows:

Supplementary table 2. The detection procedure of amplification.

| Amplification step | Cycle number | Temperature (℃) | Time (mm:ss) | Collect fluorescence |
| --- | --- | --- | --- | --- |
| 1 | 1 | 95 | 05:00 | Deny |
| 2 | 45 | 95 | 00:15 | Deny |
|  |  | 58 | 00:30 | Yes |
| 3 | 1 | 40 | 00:05 | Deny |

(3) Preparation of reagents: Remove the test strip and magnetic beads, and reheat at room temperature. Shake the magnetic beads thoroughly before use.

(4) Sample preparation: The sputum samples were liquefied before testing. 2mL sample diluent and 1mL sputum sample were homogenized in a homogenizer tube and stored.

2. Sample Testing

(1) Sample addition procedure: Open the cartridge lid, remove the opening membrane, and add 200μL ofthe test sample. Mix thoroughly by blowing. Add 10μL of magnetic beads (vibrate the mixture before use). Mix again using a pipette (it is recommended to blow from all angles ofthe pipette tube walls at least 15 times to ensure complete mixing of the magnetic beads in the liquid). Finally, close the cartridge lid.

(2) Interface operation: Select [Quick Run] on the main interface. Click the scan icon in the [Check Name] column. Align the QR code on the test cartridge with the instrument's scanning lens, then scan to launch the test program [MTBC1]. Next, click the sample information field to enter details, or tap the adjacent scan icon to scan the sample information. Then select the channel to run. When the interface prompts to insert the cartridge, insert the test cartridge with the QR code facing up, press firmly to ensure complete insertion. Click the icon in the lower right corner of the instrument to start detection. The screen displays the running interface. Tap the flip button to close the flip.

3. Data analysis

After detection is complete, the interface displays a dialog box indicating the completion status. Click the [Start Analysis] button in the lower-right corner of the screen, and the software will automatically perform data analysis. The fluorescence channels of the instrument were FAM channel (blue), ROX channel (yellow) and Cy5 channel (red).

4. Quality Control

This reagent kit incorporates an internal control (Cy5) using the human genome in the sample as an internal standard. This ensures the reliability of sample processing, nucleic acid extraction, and reagent amplification, thereby preventing false-negative results. The internal control gene must demonstrate a characteristic S-shaped amplification curve (including the initial phase before reaching the plateau) with a Ct value ≤38 for valid results. If not, the test must be repeated or a new sample collected for retesting.

Negative control: the test result software judged as "negative, negative"; The positive control was judged as "positive, positive" by the detection result software.

**Positive threshold**

The reagent kit was used to detect 240 sputum samples, including 151 male samples and 89 female samples, with an average age of 52.34±18.89 years. The positive judgment value of IS6110 and IS1081 was Ct≤38.

**Interpretation of test results**

Supplementary Table 3. Interpretation of Test Results

|  | FAM | ROX | Cy5 | Software determination | Explain |
| --- | --- | --- | --- | --- | --- |
| 1 | >38 or **∞** | >38 or **∞** | >38 or **∞** | Re-examination, reexamination | Cannot accept. Retest or resample |
| 2 | >38 or **∞** | ≤38 | >38 or **∞** | Re-examination, positive | Cannot accept. Retest or resample |
| 3 | ≤38 | >38 or **∞** | >38 or **∞** | Positive, retested | Cannot accept. Retest or resample |
| 4 | ≤38 | ≤38 | >38 or **∞** | Positive, positive | Cannot accept. Retest or resample |
| 5 | >38 or **∞** | >38 or **∞** | ≤38 | Negative, negative | Mycobacterium tuberculosis complex DNA negative, acceptable |
| 6 | ≤38 | >38 or **∞** | ≤38 | Positive, Negative | Mycobacterium tuberculosis complex DNA positive, acceptable |
| 7 | >38 or **∞** | ≤38 | ≤38 | Negative, Positive | Mycobacterium tuberculosis complex DNA positive, acceptable |
| 8 | ≤38 | ≤38 | ≤38 | Positive, positive | Mycobacterium tuberculosis complex DNA positive, acceptable |
